# Characterization of the Direct and Indirect Inhibition of Apoptosis by Full‐Length Recombinant Bcl‐xL Monomers

**DOI:** 10.1002/cbic.202500683

**Published:** 2026-01-27

**Authors:** Christina Elsner, Ludovica M. Epasto, Adeline Cieren, Dominik Gendreizig, Svetlana Kucher, Daniel Roderer, Enrica Bordignon

**Affiliations:** ^1^ Department of Physical Chemistry, Sciences II University of Geneva 30 Quai Ernest Ansermet 1211 Geneva Switzerland; ^2^ Leibniz‐Forschungsinstitut für Molekulare Pharmakologie Robert‐Rössle‐Str. 10 13125 Berlin Germany

**Keywords:** apoptosis, Bcl‐xL, electron paramagnetic resonance, large unilamellar vesicles, mitochondria

## Abstract

The Bcl‐2 protein Bcl‐xL is an inhibitor of intrinsic apoptosis which either directly inhibits the pore‐forming Bcl‐2 proteins, like Bax or Bak, or indirectly inhibits pore formation by sequestering the pro‐apoptotic BH3‐only activators. The structural basis of the inhibition of pore formation in the outer mitochondrial membrane is still largely unknown due to the lack of atomic resolution structures of the relevant inhibitory complexes at the membrane. Herein, a protocol to obtain high‐yield recombinant monomeric full‐length Bcl‐xL proteins is presented. The monomeric Bcl‐xL retains the ability to shuttle between membrane and aqueous environments and can successfully inhibit Bcl‐2‐induced membrane permeabilization via both modes of action, as proven by in vitro and *in organelle* assays with a minimal Bcl‐2 interactome constituted by Bcl‐xL, cBid, and Bax.

## Graphical abstract

Purification of full‐length Bcl‐2 proteins comes with challenges resulting in several studies being done on various truncated variants. This article presents a protocol to obtain pure monomeric full‐length Bcl‐xL, which shuttles between the membrane and aqueous environments, and retains the ability to perform the direct and indirect modes of cell death inhibition in vitro and *in* *organelle*.

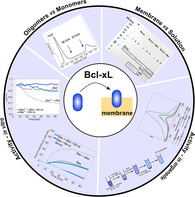

## 1 Introduction

Regulation of apoptosis plays a major role in cellular homeostasis and the immune system and any interference in the activation and inhibition mechanisms gives rise to diseases like cancer and Alzheimer's.^[^ ^1^ ^]^ In the mitochondrial pathway of apoptosis, such regulation is finely tuned by the members of the Bcl‐2 protein family. The effector or pore‐forming proteins (Bax, Bak, Bok) cause mitochondrial outer membrane permeabilization (MOMP) triggering a signal cascade ultimately leading to apoptosis. This process is activated by BH3‐only proteins (called also activators, such as cBid, Bim, PUMA, or sensitizers, such as Bad, Noxa, and so on) and inhibited by anti‐apoptotic proteins (called also pro‐survival or guardians, such as Bcl‐xL, Bcl‐2, and so on). The complex interplay between these three subgroups of the Bcl‐2 protein family, which will ultimately decide the fate of the cell, mostly requires the presence of the mitochondrial outer membrane as active player (for recent reviews see Refs. [2, 3, 4]).

The Bcl‐2 interactome is regulated by diverse interactions between Bcl‐2 homology (BH) domains and most proteins also contain a hydrophobic, putative transmembrane (TM) C‐terminal region. As their name implies, BH3‐only proteins only have the BH3‐domain and a TM C‐terminal helix (TMH) and are mostly intrinsically disordered, with the notable exception of Bid, which has a globular fold and lacks the C‐terminal TMH.^[^ ^5^ ^]^ Both pore formers and pro‐survival proteins have four conserved BH domains (1–4) and the C‐terminal TMH, the latter being often cleaved off in recombinant proteins (Δ *C* protein variants) to increase their stability in in vitro studies. However, the C‐terminal hydrophobic sequences were shown to have not only the ability to target the proteins to the membrane but also to engage in protein–protein regulatory interactions.^[^ ^4^ ^]^ Interactions between Bcl‐2 proteins are often mediated by the conserved hydrophobic groove assembled by the BH‐domains 1–3 of pore formers and pro‐survival proteins, which can efficiently bind BH3‐motifs of other Bcl‐2 proteins.^[^ ^2^ ^]^ Besides the BH3‐into‐groove interaction, BH3‐domains of proapoptotic proteins were found to bind the rear side of Bax (α1/α6 helices)^[^ ^6^ ^]^ inducing its activation. Additionally, full‐length Bax and Bcl‐xL were found to interact via α1‐helices,^[^ ^7^ ^]^ or via 3D domain swapping.^[^ ^8^ ^,^ ^9^ ^]^

The interaction between pro‐survival and pro‐apoptotic proteins is the molecular basis for the inhibition of MOMP. Three different inhibition modes have been described in literature: the guardians can regulate the localization of the effectors to reduce binding to the MOM, they can bind the pore‐formers at the membrane (direct mode), or they can sequester the BH3‐only activators (indirect mode).^[^ ^3^ ^,^ ^6^ ^,^ ^10^ ^,^ ^11^ ^]^ The latter mode is especially interesting, since it has been shown that Bcl‐xL has a higher affinity to BH3‐only proteins than to pore formers.^[^ ^2^ ^]^

**Figure 1:**
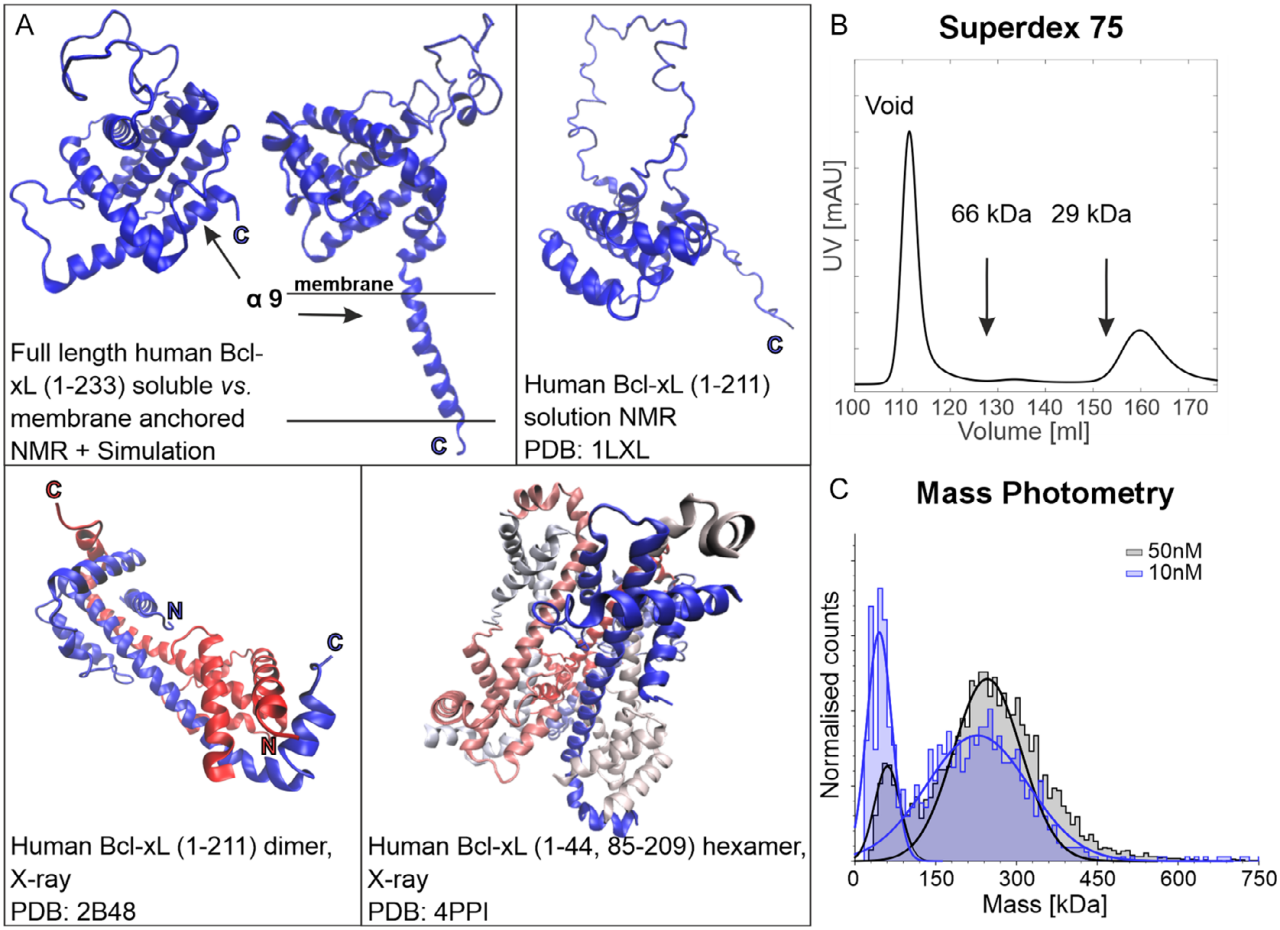
Monomeric, dimeric, and oligomeric forms of Bcl‐xL. A) Examples of Bcl‐xL structures. Upper panel left: full‐length Bcl‐xL in aqueous and nanodisc environment obtained by NMR and MD simulations (atomic coordinates kindly provided by Prof. Marassi from);^[^ ^14^ ^]^ right: NMR structure of monomeric Bcl‐xLΔ *C* in solution; lower panel left: crystal structure of a water‐soluble dimer of Bcl‐xLΔ *C* formed by 3D‐domain swapping; right: crystal structure of a water‐soluble hexamer of Bcl‐xLΔ *C*‐Δloop formed by domain‐swapped dimers. B) Size exclusion profile from our Bcl‐xL purification protocol performed on a Superdex 75 26/600 column at 4 °C. Arrows indicate reference protein elution volumes. The peak eluting in the void volume corresponds to oligomeric Bcl‐xL, named Bcl‐xL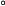 (size >70 kDa), the peak eluting at 160 ml corresponds to monomeric Bcl‐xL (molecular weight of 26 kDa). Additional SEC analysis can be found in Figure S1, Supporting Information. C) Mass photometry of Bcl‐xL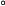 at 10 nM (blue) and 50 nM (gray) (protomer concentrations). For the 10 nM sample, we found a low‐molecular‐mass peak close to the mass sensitivity limit of the method (30 kDa) centered around 45 kDa (σ = 23 kDa, 34% fraction) and a high‐molecular‐mass peak at 228 kDa (σ = 95 kDa, 77% fraction). For the 50 nM sample, the low‐molecular‐mass peak is centered at 60 kDa (σ = 24 kDa, 14% fraction) and the high‐molecular‐mass peak at 240 kDa (σ = 82 kDa, 81% fraction).

The study of these inhibitory complexes is complicated by the challenges in preparing active, full‐length recombinant proteins at high concentrations and which can shuttle between their soluble and membrane‐bound states.^[^ ^12^ ^,^ ^13^ ^]^ Experimental atomic resolution structures of Bcl‐xL exist, but only of various deletion‐variants. In particular, the structure of Bcl‐xL in water was solved by NMR on a Δ *C* variant (PDB entry 1LXL (residues 1‐211, see **Figure** **1**A)), the BH3‐to‐groove binding was characterized on deletion‐variants of the long loop region of Bcl‐xL (e.g. Δ45–84) and of the C‐terminal TM domain (e.g. Δ212–233), in complex with peptides derived from BH3‐domains (see, e.g. PDB entries: 4QVE, 1BXL, 1G5J, 3FDL, 4CIN). Biophysical studies of full‐length Bcl‐xL in its soluble or membrane‐anchored form have shed light on the role of the C‐terminal TMH in anchoring Bcl‐xL to the membrane and binding some BH3‐only proteins (e.g. Bim), and have validated the ability of membrane‐bound Bcl‐xL to bind BH3 domains with its hydrophobic groove. **Table** **1** summarizes the available purification protocols for full‐length Bcl‐xL and the methods used to study their properties in solution and/or in membranes.

**Table 1:** Examples of available protocols for the preparation of full‐length Bcl‐xL (no PDB is available for full‐length Bcl‐xL).

Sequence length	Key purification details	Membrane interaction	Soluble protein	Activity assay	Methods and Ref.
GEF‐1‐233	1. His‐GB1‐Tag; 2. SEC	LUVs, pH‐induced membrane insertion	yes	no	CD, fluorescence^[^ ^28^ ^]^
1–233	1. C‐terminal intein‐chitin binding domain; 2. Anion‐exchange chromatography	LUVs	yes	yes	Fluorescence^[^ ^15^ ^,^ ^25^ ^]^
His‐Tag‐1‐233	1. His‐Tag; 2. SEC	no	yes	no	Purification,^[^ ^29^ ^]^ Fluorescence, EPR^[^ ^30^ ^]^
1–233	1. C‐terminal intein‐chitin binding domain; 2. Phenyl Sepharose column	LUVs and mitochondria	yes	yes	Fluorescence, e.g.^[^ ^16^ ^,^ ^19^ ^]^
1‐199‐LPETG ‐202−233	1. TM helix: His‐Tag, precipitation, solubilization; 2.Soluble domain: His‐Tag, SEC	Reconstitution of TMH and ligation of the soluble domain	no	no	NMR^[^ ^31^ ^]^
1–233	1. C‐terminal intein‐chitin binding domain; 2. Minor fraction soluble, insoluble fraction treated with urea and SDS; 3. SEC	Reconstitution into nanodiscs	yes	no	NMR and MD simulations^[^ ^14^ ^]^
1–233	1. C‐terminal intein‐chitin binding domain; 2. Anion exchange chromatography; 3. SEC	LUVs and mitochondria	yes	yes	EPR, this work

Examples exist of monomeric full‐length Bcl‐xL in solution and anchored to nanodiscs (Figure 1A),^[^ ^14^ ^]^ however truncated and full‐length Bcl‐xL have also been described to form dimers and higher order oligomers (see some examples in Figure 1A), which were previously discussed to play a potential role in apoptosis.^[^ ^8^ ^,^ ^15^, ^16^, ^17^, ^18^ ^]^

In this work, we present a simple purification protocol which allows efficient separation of full‐length Bcl‐xL monomers (residues 1–233) from the oligomeric fraction which is formed during purification (in the following named Bcl‐xL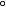). The obtained recombinant monomeric Bcl‐xL is stable and fully active, retaining the ability to shuttle between membrane and solution and to perform direct and indirect inhibition of MOMP, as demonstrated by electron paramagnetic resonance (EPR) spectroscopy and various activity assays performed on a minimal Bcl‐2 interactome in vitro and *in organelle*.

## 2 Results and Discussion

### 2.1 Characterization of Bcl‐xL: Monomers and Oligomers

In the presented protocol, we used a Bcl‐xL construct containing a cleavable C‐terminal intein tag with a chitin binding domain (Table 1), which was also successfully used in several other publications to purify full‐length Bcl‐xL (see for example).^[^ ^14^ ^,^ ^16^ ^,^ ^19^ ^]^ After affinity chromatography and tag cleavage, anion‐exchange chromatography was performed (Table 1) which was found to be necessary to achieve a high activity of the final monomeric fraction. A final size exclusion chromatography step was added to isolate the monomeric fraction from the oligomeric fraction (Figure 1B, see also Experimental Section and Figure S1–S3B, Supporting Information). The oligomeric fraction was further investigated by mass photometry and it was found to be heterogeneous in size with an average molecular weight of about 250 kDa (Figure 1C). At nanomolar concentrations, the oligomers were found to dissociate into smaller particles, likely monomers and/or dimers (Figure 1C). Negative stain electron microscopy (NS‐EM) performed on the isolated Bcl‐xL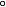 confirmed the dissociation into smaller units also at micromolar concentrations (see Figure S4, Supporting Information). The dissociation of the oligomers, and the fact that the oligomeric fraction is observed only after the anion exchange column (Figure S1 and S2, Supporting Information, also previously observed in),^[^ ^8^ ^]^ favor the hypothesis that the oligomers do not have physiological role.

### 2.2 Direct and Indirect Inhibition of Apoptosis In Vitro and *in Organelle*

**Figure 2:**
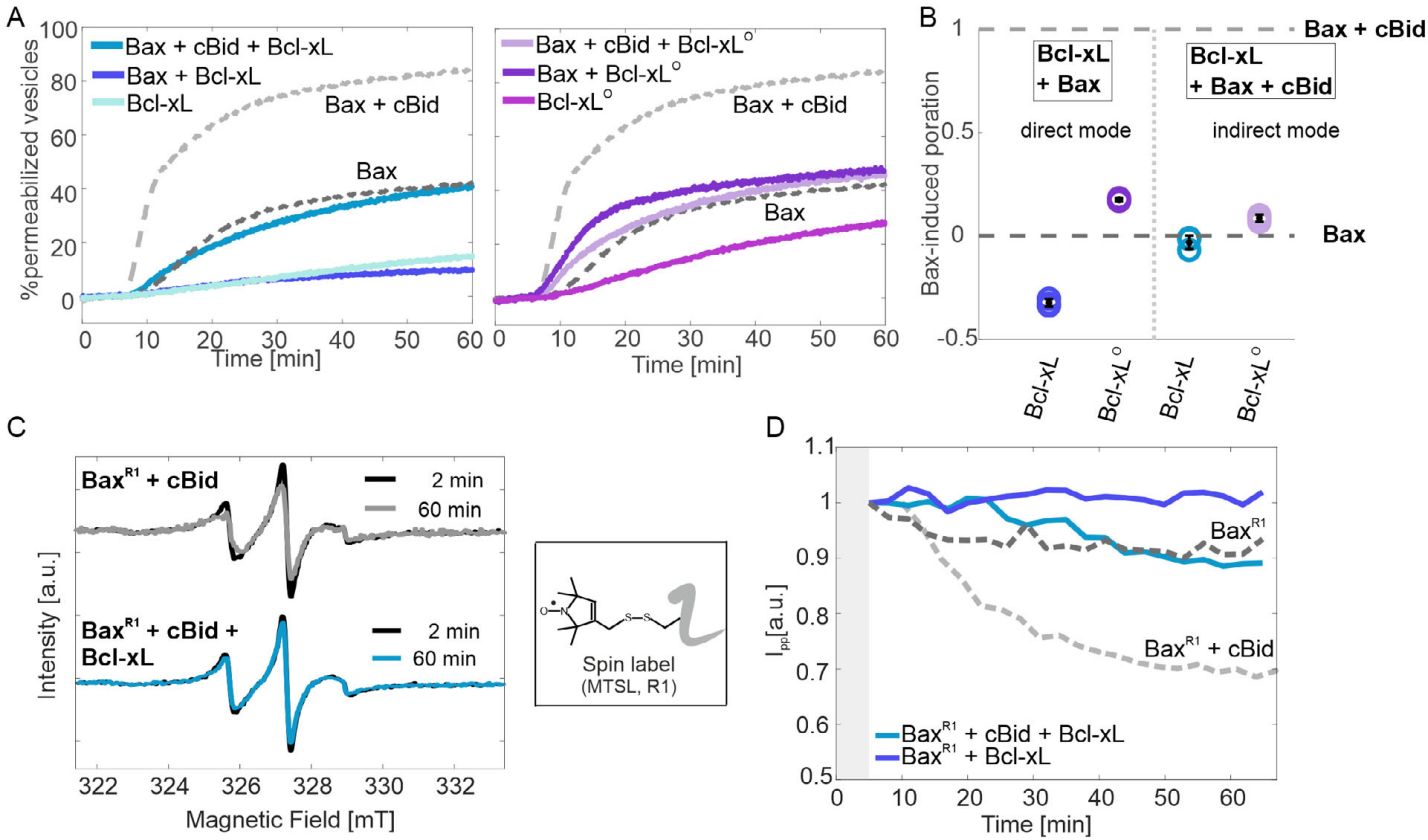
In vitro fluorescence and EPR activity assays. A) Examples of pore formation assays based on the release of the fluorescent probe calcein from LUVs through Bax‐induced pores. The time traces show the amount of permeabilized vesicles at 37 °C over time, normalized to the highest permeabilization induced by lysis with detergents (100%) (see Experimental Section and Figure S3, Supporting Information). All proteins were added at a concentration of 25 nM. The dotted gray lines on the two graphs show reference measurements (Bax alone, dark gray, 40% autoactivity; Bax + cBid, light gray, 80% activity). Left: effects induced by monomeric Bcl‐xL. Right: effects induced by Bcl‐xL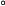. B) Comparison of three technical replicates of the inhibitory effects shown in (A). Data are taken after 30 min of incubation, the value 1 represents the normalized maximal Bax activity in presence of cBid and the value 0 represents Bax autoactivity for each replicate. C) EPR activity assay on the monomeric Bcl‐xL. Examples of the changes in EPR spectra of spin‐labeled Bax^R1^ at different incubation times at 37 °C with LUVs and cBid in absence (top) and presence of monomeric Bcl‐xL (bottom). The inset shows the spin label MTSL attached to a cysteine yielding the residue R1. The decrease of the spectral intensity reflects Bax oligomerization and membrane insertion, which is inhibited by Bcl‐xL. D) EPR kinetics of the membrane insertion of Bax^R1^ obtained by plotting the intensity of the central line of the EPR spectra (*I*_pp_) over time. Both the cBid‐induced Bax activity (light gray, dashed) and the Bax autoactivity (dark gray, dashed) are inhibited by monomeric Bcl‐xL (light and dark blue, respectively).

To further shed light on the physiological role of the two isolated fractions of Bcl‐xL, we used a minimal Bcl‐2 interactome consisting of cBid, Bax, and either monomeric Bcl‐xL or Bcl‐xL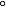. With near physiological protein concentrations (25 nM),^[^ ^2^ ^]^ we tested Bcl‐xL's ability to perform direct and indirect inhibition of Bax‐induced pore formation in vitro by monitoring the leakage of highly concentrated, self‐quenching calcein from large unilamellar vesicles (LUVs) (**Figure** **2**A,B, see also Experimental Section and Figure S3, Supporting Information). To be able to detect both modes of inhibition on the same batch of LUVs, we optimized the Bax‐to‐lipid ratio to reach a high level of autoactivity (about 40%, dark gray dashed lines in Figure 2A) while maintaining a good contrast to the maximal cBid‐induced Bax activity (Bax:cBid 1:1 stoichiometric ratio) (80% permeabilization, light gray dashed lines, in Figure 2A). Under these conditions, control experiments with Bcl‐xL monomers showed a 15% background calcein leakage, while Bcl‐xL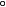 induced a higher leakage of about 30% after 60 min. We proved the direct inhibition mode by adding either monomeric or oligomeric Bcl‐xL at stoichiometric ratio in presence of Bax (1:1 protein ratio) . Monomeric Bcl‐xL efficiently inhibited Bax autoactivity to values of around 10% (blue in Figure 2A). In contrast, Bcl‐xL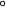 failed to inhibit Bax autoactivity (dark purple in Figure 2A). Figure 2B summarizes the results on three technical repeats. To prove the indirect inhibition mode, we added either monomeric or oligomeric Bcl‐xL at stoichiometric ratio in presence of Bax and cBid (1:1:1 protein ratio), and we found that calcein release was decreased to a value similar to Bax autoactivity (around 40%) (cyan and light purple in Figure 2A). This indicates that both Bcl‐xL monomers and the heterogeneous mixture of Bcl‐xL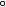 can efficiently inhibit cBid at stoichiometric ratio (for the oligomers, we consider the protomer stoichiometry) at nanomolar concentration. However, due to the heterogeneity of Bcl‐xL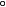 (see Figure 1C and S4, Supporting Information), it is not possible to disentangle the role of the higher order oligomers from that of the smaller units present in solution. The in vitro activity tests demonstrate that the purified monomeric full‐length Bcl‐xL retains the ability to efficiently inhibit cBid (indirect mode) as well as Bax (direct mode) at nanomolar stoichiometric ratios, while Bcl‐xL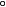 retains only the indirect mode of action.

We corroborated the activity data of the monomeric Bcl‐xL also at micromolar concentrations via EPR kinetics experiments following the membrane insertion of MTSL‐labeled wild type Bax (called for simplicity Bax$R^{1}$) (Figure 2C, D) in presence and absence of monomeric unlabeled Bcl‐xL (EPR method previously shown in).^[^ ^20^ ^,^ ^21^ ^]^ This method allows monitoring changes in the dynamics of the spin‐labeled side chains of Bax in contrast to following leakage of fluorophores from the LUVs upon pore formation. Notably, Bcl‐xL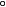 could not be tested via EPR kinetics due to the too low concentration in the stock solution. The decrease of the EPR spectral intensity (central line in Figure 2C) plotted over time (Figure 2D) indicates the reduced mobility of spin‐labeled Bax which can arise from it entering the membrane and oligomerizing either alone (autoactivity, dotted dark gray) or in presence of cBid (dotted light gray). If Bcl‐xL monomers are added at stoichiometric ratio, they inhibit Bax autoactivity (dark blue) and cBid (light blue), fully in agreement with the fluorescence data in Figure 2A, B. The fluorescence and EPR in vitro assays prove that purified monomeric full‐length Bcl‐xL inhibits Bax and cBid both at nano‐ and micromolar concentrations.

**Figure 3:**
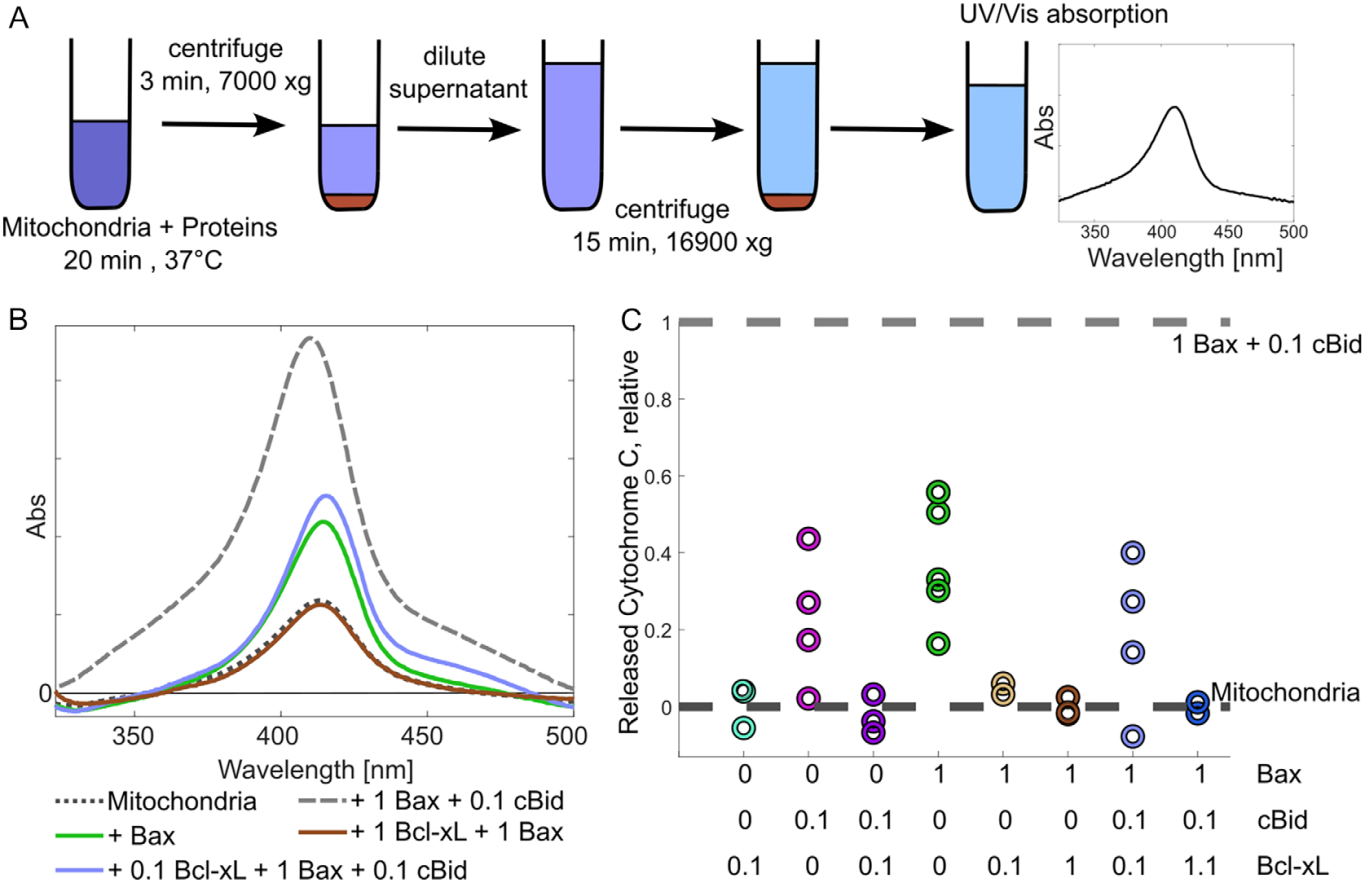
*In organelle* activity assays of monomeric Bcl‐xL. A) Schematic description of cytochrome c release assays by UV–vis spectroscopy (see Experimental Section). Mitochondria and proteins are incubated with freshly isolated mitochondria and the supernatant is then separated from the pellet via centrifugation steps. Membrane permeabilization is monitored by the maximum intensity of the UV–vis absorption of the released cytochrome c (408 and 414 nm for the oxidized and reduced form, respectively). B) Example of the background corrected spectra obtained with one mitochondria batch under different conditions. C) Statistical analysis of the cytochrome c release in presence of different combinations of proteins (biological replicates). The value 1 corresponds to the maximum release caused by incubation with Bax:cBid (1:0.1 ratio) on the mitochondria batch under consideration, the value 0 is the background release caused by incubating the respective mitochondria batch alone.

To confirm the physiological relevance of the direct and indirect modes of action of the purified monomeric Bcl‐xL, we performed activity tests using freshly isolated mitochondria from mouse liver (see **Figure** **3**A and Experimental Section). We monitored the release of cytochrome c upon incubation with Bax in presence of cBid and/or Bcl‐xL. The maximum absorption intensity of the cytochrome c released in the supernatant was used as an indicator of MOMP. Figure 3B shows examples of the UV–vis spectra obtained under different conditions (see Experimental Section) and Figure 3C the statistical analysis over several biological replicates. We found that mitochondria alone have a detectable release of cytochrome c, which we define as background release (Figure 3B and dashed dark gray line at 0 in Figure 3C) and that the maximal release is obtained in presence of Bax and cBid at 1:0.1 stoichiometric ratio (Figure 3B and dashed light gray line at 1 in Figure 3C). In contrast to the in vitro assays, cBid was not used 1:1 with Bax to minimize interaction with endogenous Bax/Bak. Bcl‐xL alone does not induce MOMP above the background level observed (cyan circles in Figure 3C), but cBid alone has a clear effect in membrane permeabilization (magenta circles in Figure 3C). The effect observed with cBid can be either due to its own ability to permeabilize membranes^[^ ^22^ ^]^ and/or to the activation of the endogenous Bax/Bak present in the mitochondria. Interestingly, when Bcl‐xL is added together with cBid, the cytochrome c release goes back to background levels (violet circles in Figure 3C), indicating that Bcl‐xL efficiently inhibits cBid‐induced MOMP. Bax alone (green circles in Figure 3C) also induces cytochrome c release, and this effect can be fully inhibited by addition of Bcl‐xL already in a 1:0.1 Bax:Bcl‐xL stoichiometric ratio (compare orange and brown circles in Figure 3C). To fully inhibit the maximal permeabilization with Bax and cBid, it was not sufficient to add Bcl‐xL at stoichiometric ratio to cBid (1:0.1:0.1, light blue circles in Figure 3C). However, a higher Bcl‐xL concentration, equal to the sum concentration of Bax and cBid, resulted in full inhibition (1:0.1:1.1 ratio, blue circles in Figure 3C). The activity assay performed in mitochondria corroborated the conclusions obtained in vitro and validated the physiological relevance of the monomeric full‐length Bcl‐xL, paving the way toward its use for biophysical analysis.

### 2.3 Water‐Membrane Partitioning of Bcl‐xL Monomers and Interactions with Bcl‐2 Partners

**Figure 4:**
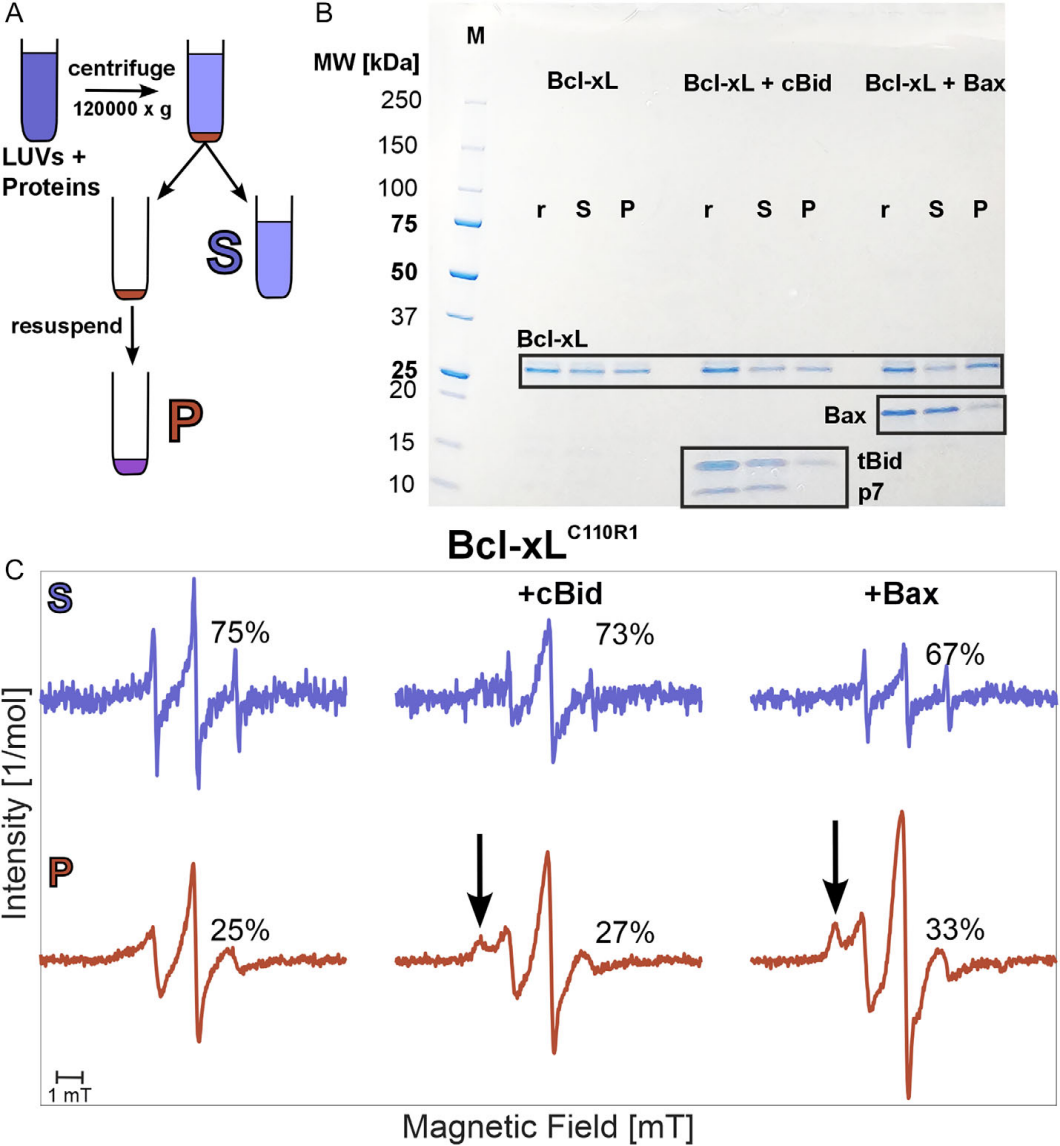
Membrane affinity of monomeric Bcl‐xL in absence and presence of partner Bcl‐2 proteins. A) Schematic description of the sample preparation. The protein–LUVs mixture is incubated for one hour at 37 °C. The pellet and supernatant fractions are separated by centrifugation (see Experimental Section). B) SDS PAGE analysis of Bcl‐xL alone or in presence of cBid or Bax and LUVs. *P*: pellet; *S*: supernatant; *r*: reference protein mixture in solution without LUVs. Boxes highlight bands corresponding to monomeric Bcl‐xL (26 kDa), Bax (21 kDa), and cBid (tBid and p7 bands at 15 and 7 kDa, respectively). C) EPR spectra of the supernatant and pellet fractions obtained from mixtures of LUVs with spin‐labeled Bcl‐xL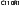 alone (left) or in combination with cBid (middle) or Bax (right) in a 1:1 stoichiometric ratio at micromolar concentration. The percentages indicate the relative amount of moles (10% estimated error) of spin‐labeled Bcl‐xL in each fraction obtained by spectral integration and taking into account the total volume of each fraction (20 μL for the pellet and about 90 μL for the supernatant). The different signal‐to‐noise ratios of the two fractions stem from the different dilution factors in the two volumes. The arrows point to spectral changes caused by the interaction of Bcl‐xL with Bcl‐2 partners in the membrane.

To understand the role of the membrane in the formation of the inhibitory complexes, we characterized the partitioning of Bcl‐xL between aqueous solution and membrane environment using sodium dodecyl sulfate–polyacrylamide gel electrophoresis (SDS PAGE) and EPR spectroscopy. In addition, we studied the effect exerted on the water‐membrane equilibrium of Bcl‐xL by other Bcl‐2 partners, namely cBid and Bax. To quantify the amount of Bcl‐xL in both environments, we used a simple centrifugation method: we incubated Bcl‐xL and LUVs in the presence or absence of cBid or Bax for one hour at 37 °C, followed by separation of the mixture via centrifugation (**Figure** **4**A). The pellet (P) and supernatant (S) fractions were separately analyzed (Figure 4B,C).

The SDS PAGE analysis shown in Figure 4B confirms the equilibrium of Bcl‐xL between its membrane‐bound and soluble states, corroborating previous data *in vivo*, showing that about 50% Bcl‐xL is cytoplasmic.^[^ ^12^ ^]^ The presence of cBid does not affect this equilibrium. Notably, in the supernatant both bands of the cleaved cBid (tBid, 15 kDa and p7, 7 kDa) can be detected, however, the pellet fraction shows only tBid. If full‐length Bax is present, the amount of Bcl‐xL at the membrane slightly increases and most of Bax is found in the supernatant.

To investigate Bcl‐xL in solution and in membrane by EPR, we engineered an accessible cysteine at position 110 and spin labeled it with MTSL (Bcl‐xL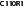). After labeling, the proteins retain their activity, as proven by pore formation assays (see Figure S3, Supporting Information). The label is strategically positioned in the hydrophobic groove of Bcl‐xL, which was previously shown to bind BH3‐peptides.^[^ ^8^ ^,^ ^17^ ^]^ The EPR spectra of Bcl‐xL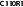 recorded in the supernatant and pellet fractions allowed quantification of the labeled proteins using the double integral of the spectra, proportional to the concentration of Bcl‐xL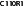 (Figure 4C). We found that 25% of the total amount of Bcl‐xL monomers binds to the membrane. This partitioning remains unchanged in the presence of cBid. In the presence of Bax, the amount of membrane‐bound Bcl‐xL slightly increased to 33%, in line with the result from SDS PAGE (Figure 4B). Notably, the spectral shape of Bcl‐xL in all three supernatants remained similar to that of the protein in solution (see Figure S5, Supporting Information). However, the spectra of Bcl‐xL in the membrane changed when cBid or Bax were present during incubation. The arrows in Figure 4C highlight the broad spectral component emerging in the presence of the Bcl‐2 partners, which indicates that the motion of the spin‐labeled side chain in the hydrophobic groove of Bcl‐xL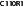 is slowed down due to sterical hindrance, possibly caused by a direct interaction with the other Bcl‐2 partners (formation of hetero‐complexes) and/or by structural changes within Bcl‐xL triggered by the presence of the interaction partner.

## 3 Conclusion

The presented optimized purification protocol of recombinant full‐length Bcl‐xL yielded a pure, fully active water‐soluble monomeric Bcl‐xL well separated from a fraction of higher order oligomers. The oligomers, which are formed during the purification procedure, easily aggregate upon concentration, partially dissociate into smaller units at nanomolar concentrations, and can execute only the indirect mode of inhibition. The propensity of truncated variants of Bcl‐xL to form dimers and higher‐order oligomers and the difficulty in avoiding large oligomers when purifying full‐length Bcl‐xL prompted speculations about their possible role in the inhibition of apoptosis. Notably, it was shown for the 3D domain‐swapped dimer formed by truncated Bcl‐xL variants, that the BH3‐binding groove remains accessible to BH3 peptides,^[^ ^17^ ^]^ which might be sufficient to retain the ability to indirectly inhibit pore formation via sequestration of BH3‐only proteins. Here, we found that the oligomers can also target the membrane (Figure S6, Supporitng Information), which will possibly facilitate the interaction with tBid, explaining the observed indirect inhibition pathway.

In contrast, the isolated monomeric fraction is stable, and can be concentrated up to 10–30 μM, which is favorable for further biophysical studies. Monomeric Bcl‐xL is proven to execute both direct and indirect modes of inhibition, as shown by assays using a minimal interactome consisting of Bcl‐xL, cBid, and Bax. We used established in vitro pore formation assays, which provides standardized activity tests of protein batches and complemented them with *in organelle* assays that have a higher physiological relevance, still allowing fast detection of the activity of different combinations of Bcl‐2 proteins.

With this protocol, we obtained recombinant fully active proteins at micromolar concentrations, which we could spin label without loss of activity. This paves the way toward the use of site‐directed spin labeling EPR and other biophysical methods to study dynamics and structures of inhibitory complexes at the membrane. Indeed, we identified a dynamic change in the hydrophobic groove of membrane‐embedded spin‐labeled Bcl‐xL not only in presence of tBid but also in the presence of Bax, which is promising for the further elucidation of the structural basis of the regulatory mechanisms of Bcl‐2 proteins in the mitochondrial pathway of apoptosis.

## 4 Experimental Section

4.1

4.1.1

##### Expression of Recombinant Proteins

The plasmids pTYB1‐Bcl‐xLC110 (S110C‐C151S) and pET23d‐BidC82 (C30S‐S82C‐C126S) were cloned by site‐specific mutagenesis and controlled via DNA sequencing. His‐tagged mouse Bid (pET23d),^[^ ^23^ ^]^ human Bax (pTYB1),^[^ ^24^ ^]^ and human Bcl‐xL (pTYB1),^[^ ^25^ ^]^ kindly provided by Prof. Garcia‐Saez, were expressed in *E. coli* and induced with IPTG for 4 h (Bid) or overnight (Bcl‐xL, Bax) and the pellets were either used immediately or shock frozen in liquid nitrogen and stored at −80 °C. After lysis of the bacteria in a microfluidizer and incubation with 1‐200U DNase I per liter of bacterial culture, the membranes and intact bacteria were separated from the soluble fraction via centrifugation at 35000xg for 35 min.

##### Purification and Cleavage of Bid

Bid was purified from the soluble fraction of the bacterial lysate using Ni‐NTA (Ni‐NTA Superflow by Qiagen). It was washed first with Nickel buffer (20 mM Tris, pH 7.5, 20 mM NaH2PO4, 300 mM NaCl) without imidazole, then with 10 and 25 mM before being eluted with 250 mM imidazole. The protein was stored in storage buffer (20 mM Tris, pH 7.5, 150 mM NaCl).

Bid (100–150 µM) was mixed 1:1 with cleavage buffer (50 mM HEPES, pH 7.5, 100 mM NaCl, 10 mM DTT, 1 mM EDTA, 10% sucrose) and Caspase 8 (final concentration 1 μM). The mixture was incubated for 3 h at room temperature and purified using Ni‐NTA, washed with Nickel buffer without imidazole, eluted with 250 mM imidazole, and dialyzed back into storage buffer. The concentration was determined via absorption at 280 nm $\left(\varepsilon_{Bid}=8490\frac{1}{Mcm}\right)$ using a DeNovix DS‐11 FX UV–vis spectrometer. In the following, the cleaved Bid is called cBid, the 15 kDa fragment of cBid is called tBid (also known as p15 in literature) and the 7 kDa fragment is called p7.

##### Purification and Spin Labeling of Bcl‐xL and Bax

Bcl‐xL and Bax were purified from the soluble fraction of the bacterial lysate using a chitin resin (New England Biolabs) which was washed 20× with chitin buffer (20 mM Tris (Bcl‐xL) or 8 mM Tris (Bax), pH 8.0, 500 mM NaCl) and 3x cleavage buffer (chitin buffer + 30 mM DTT freshly added). The column was filled with cleavage buffer, closed, and incubated at 4 °C overnight to cleave off the intein tag. The proteins were eluted with chitin buffer and dialyzed 1:10 against 20 mM Tris (pH 8.0) with one step (Bcl‐xL) or 8 mM Tris (pH 8.0) in five steps (Bax), respectively.

For Bcl‐xL, 1 mM DTT was added to the dialysate before loading on an anion exchange column (POROS GoPure 50 HQ Pre‐packed Column) at 0.5 ml min^−1^. The column was washed with Mono A50 buffer (20 mM Tris, pH 8.0, 50 mM NaCl, 1 mM DTT) and the protein was eluted running a gradient with Mono B buffer (20 mM Tris, pH 8.0, 1 M NaCl, 1 mM DTT). The pooled fractions of the main peak were injected into a size exclusion column (Superdex 200 10/300) equilibrated with storage buffer. If the protein had to be labeled afterwards, 10 μM TCEP was freshly added to the storage buffer. The resulting oligomer peak was pooled but not concentrated as centrifugal filters cause the oligomers to aggregate. The monomer peak was pooled and concentrated in centrifugal filters (Vivaspin Turbo 4 Sartorius (cutoff 10 kDa)) (see Figure S1, Supporting Information). During the optimization procedure, we found that skipping the anion exchange column and performing directly the SEC resulted in significantly higher amount of monomers with respect to oligomers; however the resulting monomers were less active (see Figure S2 and S3, Supporting Information).

Starting from a culture of 1.5 liters, we collected 4 mL of the monomeric fraction at a concentration of around 1–2 μM, which was found to be stable in buffers at pH 7.5 and could be further concentrated in centrifugal units to 10–30 µM with negligible protein losses. The Bcl‐xL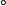 fraction (10 mL, 5–10 μM protomer concentration) could not be further concentrated due to severe protein aggregation. Concentrations were determined via absorption at 280 nm ($\varepsilon_{Bcl-xL}=47440\frac{1}{Mcm}$) using a DeNovix DS‐11 FX UV–vis spectrometer.

To label the monomeric fraction of Bcl‐xL, 5–10x excess MTSL was added per protein (protein concentration 10–20 μM) and incubated overnight at 8 °C. The excess label was removed by washing 4–5x in centrifugal filters (Vivaspin Turbo 4 Sartorius (cutoff 10 kDa)) with storage buffer. The spin labeling efficiency (spin per cysteine) of monomeric Bcl‐xL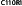 was 49%, as calculated by comparing the second integral of the cw EPR spectra with a reference TEMPOL (T) standard (see Figure S5, Supporting Information).

(1) $$c(\mathrm{spin})=\frac{c(T)}{\int \int (T)}\cdot \iint (\mathrm{spin})$$

For Bax, the dialysate was loaded on an anion exchange column (HiTrap Q High Performance) at 0.5 ml min^−1^. The column was washed with Mono A buffer (8 mM Tris, pH 8.0) and the protein was eluted running a gradient with Mono B buffer (20 mM Tris, pH 8.0, 1 M NaCl) pooling the first peak. Concentrations were determined via absorption at 280 nm $\left(\varepsilon_{\mathrm{Bax}}=36940\frac{1}{\mathrm{Mcm}}\right)$ using a DeNovix DS‐11 FX UV–vis spectrometer.

To label wild type Bax (containing two natural cysteines C62 and C126), 10x excess MTSL per protein (protein concentration 10–20 μM) was added and incubated overnight at 4 °C. The excess label was removed by washing 4–5x in centrifugal filters (Vivaspin Turbo 4 Sartorius (cutoff 10 kDa)) with storage buffer. The spin labeling efficiency of Bax^R1^ was 94% (spin per cysteine), as calculated by comparing the second integral of the cw EPR spectra with a reference TEMPOL standard (see Figure S5, Supporting Information).

For both, Bax and Bcl‐xL, we found that using the Vivaspin Turbo 4 Sartorius centrifugal filters caused less protein loss compared to other centrifugal filters. For Bcl‐xL, removing excess label in spin desalting columns resulted in complete protein loss.

All proteins were tested in pore formation assays before further experimental use.

##### Mass Photometry

Mass photometry was measured at different concentrations on a TwoMP Mass Photometer from Refeyn (mass range 30 kDa–5 MDa) in the Protein Production and Structure Core Facility at EPFL, Lausanne, Switzerland.

##### LUVs Preparation

The lipid mixture mimicking the mitochondrial outer membrane was composed of 46% egg L‐α‐phosphatidyl choline (PC), 25% egg L‐α phosphatidyl ethanolamine (PE), 11% bovine liver L‐α‐phosphatidyl inositol (PI), 10% 18:1 phosphatidyl serine (PS), and 8% cardiolipin (CL) (% w/w). The lipid mixture in chloroform was aliquoted, dried under vacuum, flushed with nitrogen, and stored at −80 °C for a maximum time of one year. To prepare large unilamellar vesicles, the mix was solved in either storage buffer to give a stock solution of 20 mg ml^−1^ (for EPR experiments) or in calcein (80 mM, adjusted to pH 7.0 with NaOH, Sigma) (pore formation assays) using five freeze‐and‐thaw cycles and extruded 41x through a membrane with 400 nm pores. For the pore formation assays, the LUVs were passed through a desalting column (Econo‐Pac 10DG Desalting Columns, Biorad) equilibrated in outside buffer (20 mM HEPES, pH 7.0, 140 mM NaCl, 1 mM EDTA) to remove nontrapped calcein and diluted to a final maximum concentration of 1.25 mg ml^−1^.

##### Pore Formation Assays in LUVs

Pore formation assays were performed at 37 °C in outside buffer with maximum 0.02 mg ml^−1^ LUVs and protein concentrations of 25 nM. The fluorescence was monitored over time (excitation: 495 nm, emission: 520 nm), observing the fluorescence increase of the calcein trapped in LUVs due to the dequenching after being released in the surrounding buffer upon pore formation. To determine the maximum fluorescence (100% value), triton‐X‐100 (max. 0.05%) was added at the end of the fluorescence kinetic measurement. In all protein combinations, the inhibitor (Bcl‐xL) was added first, then the pore former (Bax) and finally, the activator (cBid) in a 1:1:1 stoichiometric ratio.

##### Mitochondria Purification and Cytochrome c Release Assays

Crude mitochondria were isolated from mouse liver as described in literature.^[^ ^26^ ^]^ In short, fresh mouse livers with a total of ≈3.2 g were cut in small pieces on ice and homogenized in sucrose buffer using a glass dounce homogenizer. The homogenate was centrifuged at 600 × g for 10 minutes at 4 °C to remove any cell debris. Subsequentially, the supernatant was centrifuged at 7000 × g for 10 min at 4 °C. ≈1 mL of crude mitochondria suspension in sucrose buffer was then obtained and kept on ice. Mitochondrial proteins were considered as an index of mitochondrial concentration, and they were determined using a Bradford assay. The final protein concentration for mitochondria extraction amounted around 90 mg mL^−1^.

For mitochondria extraction different mouse genotypes were used depending on the availability: 1) IL‐33 floxed, 1x CAS‐9. 2) C57BL/6 SOPF. 3) Etv1‐CreERT2. 4) SALSA(Stim1; Stim2 dKo) CEBP‐Cre. 5) ERMP1.

In order to test the direct and indirect inhibition of Bax activation by Bcl‐xL, cytochrome c release assays were performed. Bax (7 μM), cBid (0.85 μM), and/or Bcl‐xL (0.85 μM) were mixed on ice with an aliquot of mitochondria suspension containing 23 ± 5mg mL^−1^ of proteins. To avoid mitochondrial disruption due to osmotic damage, the ratio between the protein buffer and the sucrose buffer was kept as 1:1. The samples were incubated at 37 °C for 20 min and centrifuged for 3 min at 7000 × g and 4 °C. Then, 20 μL of supernatant were diluted with 40 μL of sucrose buffer and centrifuged for 15 min at 16,900 × g and 4 °C.

The absorbance spectrum of the supernatant was collected between 260 and 800 nm. In order to evaluate the absorbance of the Soret peak, the spectra were background corrected by an exponential fit (see Figure S3, Supporting Information). The data processing was performed on MATLAB R2023b. For statistical analysis the maximum of the traces was used instead of a specific wavelength due to absorption shifts caused by the ratio of oxidized versus reduced cytochrome c.^[^ ^27^ ^]^

##### Pellet and Supernatant Preparation

All pellet/supernatant samples analyzed (SDS PAGE, cw EPR) have been prepared as described in Figure 4A. Proteins and LUVs were mixed with a final protein concentration of 10–20 μM and a final lipid concentration of 1–5 mg ml^−1^. After one hour incubation at 37 °C, the membrane and water sample were separated via centrifugation (120000 × g). The pellet was resuspended and adjusted according to the methods requirements. For the cw EPR measurements, the samples were prepared at an initial volume of 100 μL. The pellet was resuspended to a total volume of 20 μL while the supernatant volume was around 90 μL.

##### Continuous Wave (cw) EPR

All cw EPR experiments were performed on a Bruker E500 X‐band equipped with a super high Q cavity ER 4122 SHQ with a power of 2 mW and a modulation amplitude of 0.1 or 0.15 mT.

For the time‐resolved measurements, four spectra were averaged before moving to the next time point. The temperature was controlled at 37 °C with a nitrogen flow cryostat and a temperature controller (Eurotherm). Samples were measured in a total volume of 20 μL in glass capillaries (Blaubrand). Proteins at 10–20 μM final concentrations were mixed on ice and introduced into the glass capillary. In a second step, a small volume of LUVs was added to the capillary. The first spectrum was detected after 2.5 min from the addition of LUVs to the protein mixture.

##### NS‐EM and Data Analysis

3 μL of oligomeric Bcl‐xL (0.05 mg ml^−1^) were applied to a freshly glow discharged 400‐mesh carbon‐coated copper grid and incubated for 1 min before staining with 0.75% uranyl formate solution for 1 min. Grids were air dried for 5 min before they were imaged in a J1400 TEM (Jeol) equipped with a TVIPS 4k x 4k F416 detector.

36 micrographs recorded at 80,000x magnification (pixel size: 1.36 Å/px) were imported in CryoSPARC (version 4.7.0) and processed in negative stain mode with constant CTF. Particles were identified using the blob picker set to a diameter between 70 and 150 unicodex212B. 3,664 particles were extracted at a pixel size 2.72 Å/px and subjected to 2D classification using 36 classes to distinguish between intact oligomers and monomers.

## Conflict of Interest

The authors declare no conflict of interest.

## Supporting information

Supplementary Material

## Data Availability

The data that support the findings of this study are available from the corresponding author upon reasonable request.

## References

[1] P. E. Czabotar , G. Lessene , A. Strasser , J. M. Adams , Nat. Rev. Mol. Cell Biol. 2014, 15, 49. 24355989 10.1038/nrm3722

[2] J. Kale , E. J. Osterlund , D. W. Andrews , Cell Death Differ. 2018, 25, 65. 29149100 10.1038/cdd.2017.186 PMC5729540

[3] P. E. Czabotar , A. J. Garcia‐Saez , Nat. Rev. Mol. Cell Biol. 2023, 24, 732. 37438560 10.1038/s41580-023-00629-4

[4] D. Nguyen , E. Osterlund , J. Kale , D. W. Andrews , Biochem. J. 2024, 481, 903. 38985308 10.1042/BCJ20210352 PMC11346437

[5] L. P. Billen , A. Shamas‐Din , D. W. Andrews , Oncogene 2008, 27, S93. 19641510 10.1038/onc.2009.47

[6] D. Westphal , R. M. Kluck , G. Dewson , Cell Death Differ. 2014, 21, 196. 24162660 10.1038/cdd.2013.139 PMC3890949

[7] J. Ding , B. H. Mooers , Z. Zhang , J. Kale , D. Falcone , J. McNichol , B. Huang , X. C. Zhang , C. Xing , D. W. Andrews , J. Lin , J. Biol. Chem. 2014, 289, 11873. 24616095 10.1074/jbc.M114.552562 PMC4002096

[8] J. W. O’Neill , M. K. Manion , B. Maguire , D. M. Hockenbery , J. Mol. Biol. 2006, 356, 367. 16368107 10.1016/j.jmb.2005.11.032

[9] P. E. Czabotar , D. Westphal , G. Dewson , S. Ma , C. Hockings , W. D. Fairlie , E. F. Lee , S. Yao , A. Y. Robin , B. J. Smith , D. C. S. Huang , R. M. Kluck , J. M. Adams , P. M. Colman , Cell 2013, 152, 519. 23374347 10.1016/j.cell.2012.12.031

[10] F. Llambi , T. Moldoveanu , S. W. G. Tait , L. Bouchier‐Hayes , J. Temirov , L. L. McCormick , C. P. Dillon , D. R. Green , Mol. Cell 2011, 44, 517. 22036586 10.1016/j.molcel.2011.10.001 PMC3221787

[11] H. Kalkavan , D. R. Green , Cell Death Differ. 2018, 25, 46. 29053143 10.1038/cdd.2017.179 PMC5729535

[12] A. Aranovich , Q. Liu , T. Collins , F. Geng , S. Dixit , B. Leber , D. W. Andrews , Mol. Cell 2012, 45, 754. 22464442 10.1016/j.molcel.2012.01.030

[13] Y.‐T. Hsu , K. G. Wolter , R. J. Youle , Proc. Nat. Acad. Sci. 1997, 94, 3668. 9108035 10.1073/pnas.94.8.3668 PMC20498

[14] P. Ryzhov , Y. Tian , Y. Yao , A. A. Bobkov , W. Im , F. M. Marassi , Biophys. J. 2020, 119, 1324. 32888404 10.1016/j.bpj.2020.08.014 PMC7567986

[15] S. Bleicken , A. Hantusch , K. K. Das , T. Frickey , A. J. Garcia‐Saez , Nat. Commun. 2017, 8, 73. 28706229 10.1038/s41467-017-00086-6 PMC5509671

[16] C. Bogner , J. Kale , J. Pogmore , X. Chi , A. Shamas‐Din , C. Fradin , B. Leber , D. W. Andrews , Mol. Cell 2020, 77, 901. 32001105 10.1016/j.molcel.2019.12.025

[17] S. Rajan , M. Choi , Q. T. Nguyen , H. Ye , W. Liu , H. T. Toh , C. Kang , N. Kamariah , C. Li , H. Huang , C. White , K. Baek , G. Grüber , H. S. Yoon , Sci. Rep. 2015, 5, 10609. 26023881 10.1038/srep10609 PMC4448555

[18] A. Y. Denisov , T. Sprules , J. Fraser , G. Kozlov , K. Gehring , Biochemistry 2007, 46, 734. 17223694 10.1021/bi062080a

[19] J. Kale , X. Chi , B. Leber , D. Andrews , Methods in Enzymology. Vol. 544, (Elsevier, 2014), San Diego, CA, pp. 1–23. 24974284 10.1016/B978-0-12-417158-9.00001-7

[20] S. Bleicken , M. Classen , P. V. L. Padmavathi , T. Ishikawa , K. Zeth , H.‐J. Steinhoff , E. Bordignon , J. Biol. Chem. 2010, 285, 6636. 20008353 10.1074/jbc.M109.081539 PMC2825459

[21] S. Bleicken , T. E. Assafa , C. Stegmueller , A. Wittig , A. J. Garcia‐Saez , E. Bordignon , Cell Death Differ. 2018, 25, 1717. 30185826 10.1038/s41418-018-0184-6 PMC6180131

[22] H. Flores‐Romero , L. Hohorst , M. John , M.‐C. Albert , L. E. King , L. Beckmann , T. Szabo , V. Hertlein , X. Luo , A. Villunger , L. P. Frenzel , H. Kashkar , A. J. Garcia‐Saez , Embo J. 2022, 41, e108690. 34931711 10.15252/embj.2021108690 PMC8762556

[23] S. Desagher , A. Osen‐Sand , A. Nichols , R. Eskes , S. Montessuit , S. Lauper , K. Maundrell , B. Antonsson , J.‐C. Martinou , J. Cell Biol. 1999, 144, 891. 10085289 10.1083/jcb.144.5.891 PMC2148190

[24] M. Suzuki , R. J. Youle , N. Tjandra , Cell 2000, 103, 645. 11106734 10.1016/s0092-8674(00)00167-7

[25] S. Bleicken , C. Wagner , A. J. García‐Sáez , Biophys. J. 2013, 104, 421. 23442864 10.1016/j.bpj.2012.12.010 PMC3552256

[26] C. Frezza , S. Cipolat , L. Scorrano , Nat. Protoc. 2007, 2, 287. 17406588 10.1038/nprot.2006.478

[27] R. Schweitzer‐Stenner , New J. Sci. 2014, 2014, 1.

[28] G. R. Thuduppathy , J. W. Craig , V. Kholodenko , A. Schon , R. B. Hill , J. Mol. Biol. 2006, 359, 1045. 16650855 10.1016/j.jmb.2006.03.052 PMC1785297

[29] M. Vargas‐Uribe , M. V. Rodnin , A. S. Ladokhin , Biochemistry 2013, 52, 7901. 24134052 10.1021/bi400926k PMC3882133

[30] V. Vasquez‐Montes , M. Vargas‐Uribe , N. K. Pandey , M. V. Rodnin , R. Langen , A. S. Ladokhin , Biochim. Biophys. Acta (BBA) ‐ Proteins Proteomics 2019, 1867, 691. 31004798 10.1016/j.bbapap.2019.04.006 PMC6589348

[31] K. Raltchev , J. Pipercevic , F. Hagn , Chem. Euro. J. 2018, 24, 5493. 10.1002/chem.201800812 29457664

