# Supplementary material for: Characterization of the Direct and Indirect Inhibition of Apoptosis by Full‐Length Recombinant Bcl‐xL Monomers
Source: Chembiochem. 2026 Jan 27;27(2):e202500683. doi: 10.1002/cbic.202500683 (PMC12836140; doi:10.1002/cbic.202500683)
Supplement: Supplementary file 1 — Supplementary Material [file CBIC-27-e202500683-s001.pdf]

Supplementary Information

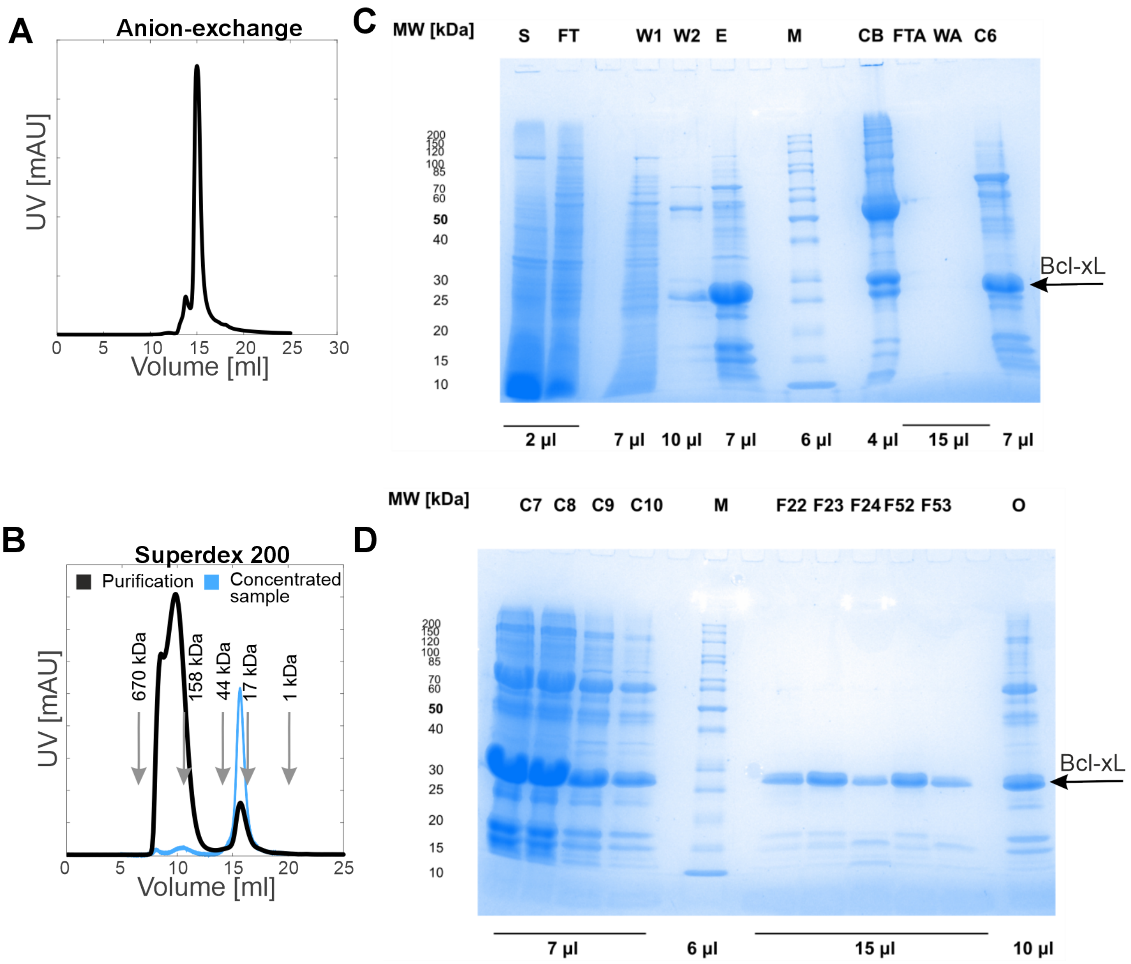

Figure S1: Bcl-xL 3-step purification. A: Anion-exchange chromatography detected at 280 nm during the Bcl-xL WT purification. The highest peak was pooled (2 ml total, fractions B6-B3) and used for further steps. SDS PAGE samples were prepared from all pooled fraction and the two neighboring fractions (B7 and B2) **B:** Black: SEC analysis of the main peak eluted from the anion-exchange during Bcl-xL purification using a Superdex 200 10/300. Since the maximum sample volume was 1 ml, this was run twice and the peaks considered together. Blue: SEC analysis of the concentrated Bcl-xL monomer (sample: 50 μl, 52 μM) using a Superdex 200 10/300. Absorption detected at 280 nm. The oligomer peaks were flash frozen and stored at -80°C, the monomer fractions were pooled, concentrated, aliquoted, and flash frozen before storage at -80°C. **C, D:** SDS PAGE analysis of the purification. S: Supernatant after cell-lysis. FT: flow through of the cell lysate loading onto the chitin beads. W1, W2: washing steps of the chitin beads. E: Elution of Bcl-xL WT off of the chitin beads after tag cleavage. M: Ladder. CB: Analysis of the chitin beads after elution but before cleaning. FTA: Flow through of the anion-exchange column. WTA: Wash of the anion exchange column. C6-C10: Fractions collected during the anion exchange. F22-24 + F52-F53: Monomer fractions after SEC and before pooling and concentrating. O: Pooled oligomer fractions. SDS PAGE samples were mixed with Laemmli buffer and heated to 95°C for 5 minutes. No reducing agents were used. Details of the purification are described in materials & methods.

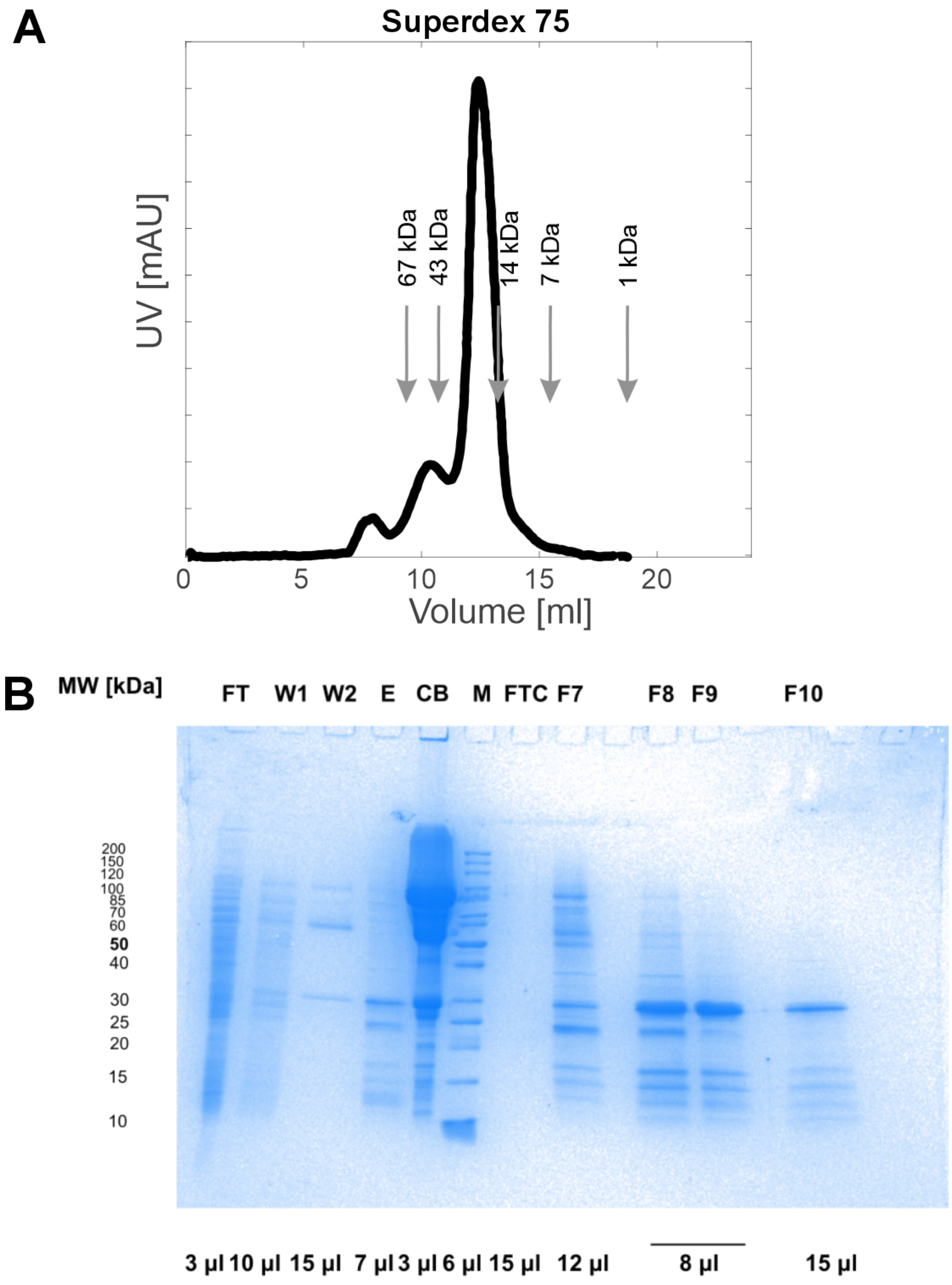

Figure S2: Bcl-xL 2-step purification (without anion exchange column). A: SEC of the concentrated chitin bead eluate on a Superdex 75 10/300 with indicated calibration. Absorption detected at 280 nm. The main peak was pooled, aliquoted, and flash frozen before storing at -80°C. SDS PAGE samples were taken from the main peak and the two neighboring fractions. **B:** SDS PAGE analysis of the purification. FT: flow through of the cell lysate loading onto the chitin beads. W1 + W2: washing steps of the chitin beads. E: Elution of Bcl-xL WT off of the chitin beads after tag cleavage. The 45 ml of eluate were concentrated to 1 ml for SEC analysis. CB: Analysis of the chitin beads after elution but before cleaning. M: Ladder. F7-F10: Fractions collected of the SEC. SDS PAGE samples were mixed with Laemmli buffer and heated to 95°C for 5 minutes. No reducing agents were used.

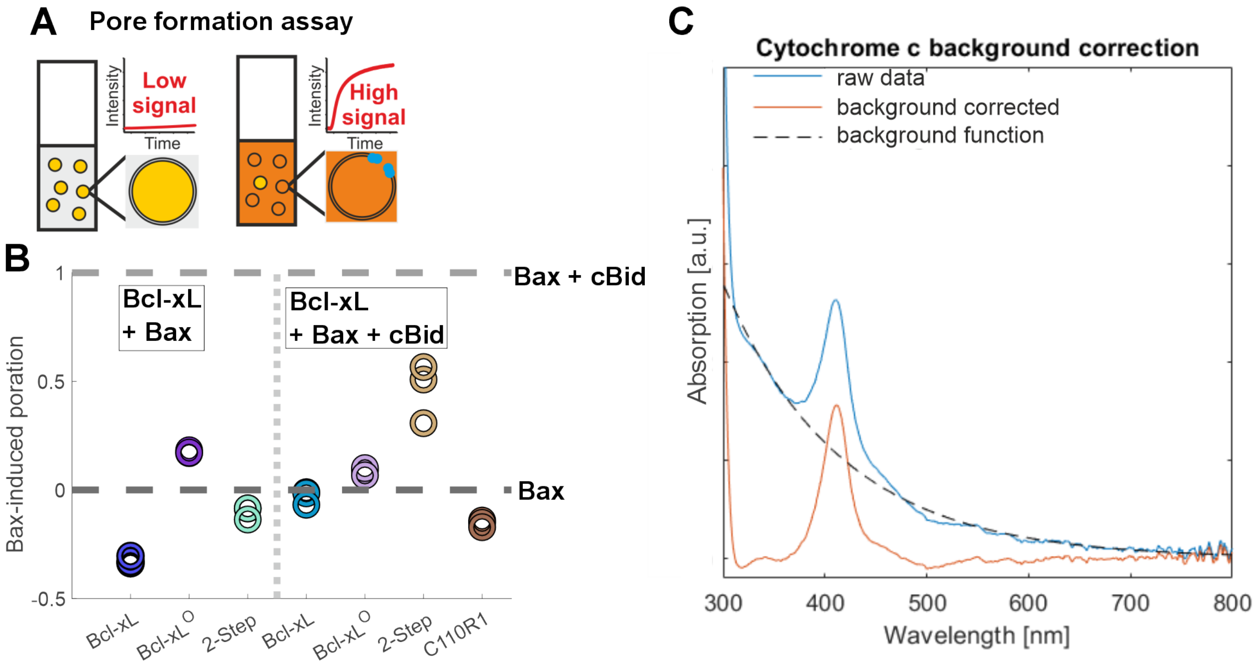

Figure S3: Activity assays. A: Graphic explanation of *in vitro* pore formation assays. Adapted from [doi: 10.1038/s41598-019-49370-z]. When the calcein is trapped at high concentration (80 mM) in the LUVs, it self-quenches leading to a low fluorescence signal. Upon membrane permeabilization by Bax, calcein is diluted in the surrounding buffer, leading to an increase in fluorescence. **B:** Comparison of technical replicates of the inhibitory effects of different Bcl-xL variants after 30 min of incubation: 1 is the normalized maximal Bax activity in presence of cBid, 0 is Bax autoactivity. Bcl-xL and Bcl-xL^O are monomers and oligomers (see fig. 2) as prepared in the 3-step purification described in materials & methods and in fig. S1. Following the same protocol, also the Bcl-xLC110R1 labeled monomer variant was prepared. The sample named '2-Step' is the monomer prepared in a 2-step purification as shown in fig. S2 showing a decreased inhibition of the Bax/cBid mixture as compared to the other monomers prepared with a 3-step purification. **C:** Example of background correction of the cytochrome c UV-vis spectrum with a monoexponential background function.

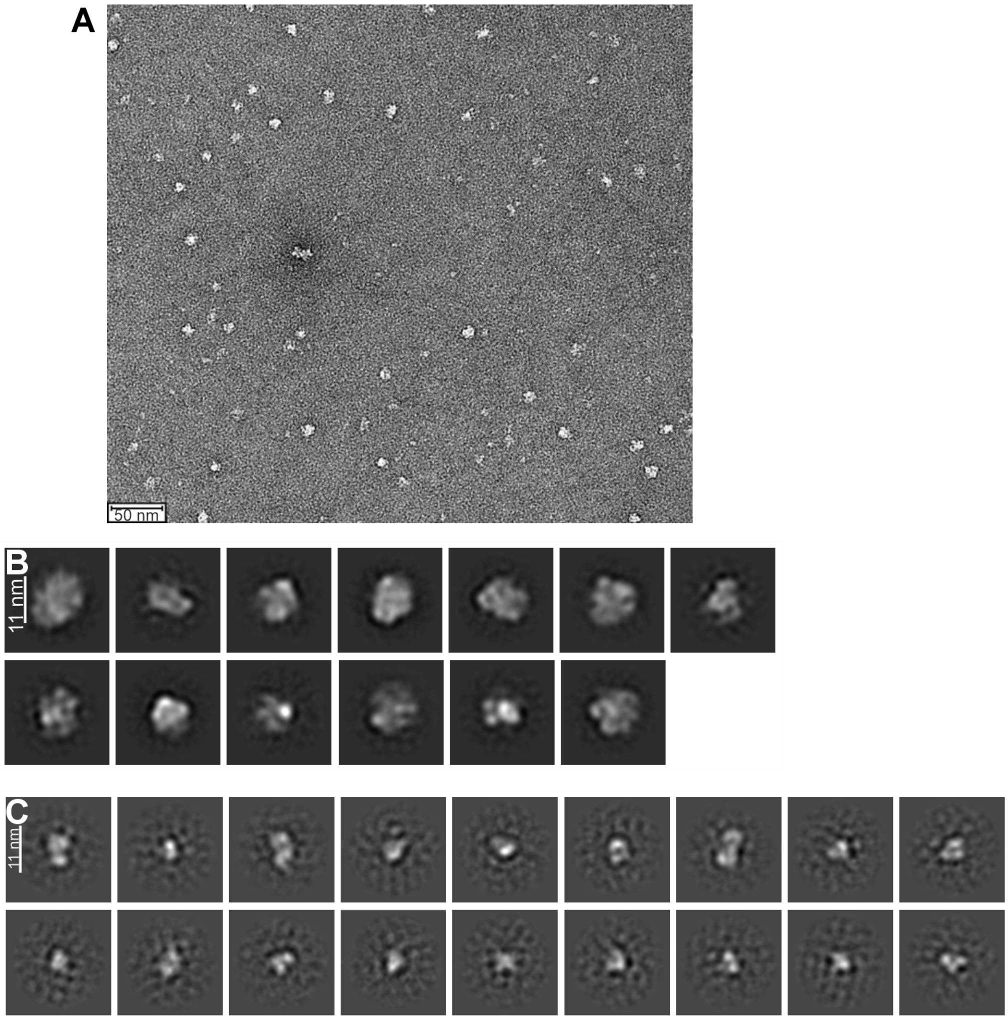

Figure S4: **A:** Representative EM micrograph of negatively stained of Bcl-xL^{O}. **B:** 2D class averages of 1712 particles of negative stain data obtained on Bcl-xL^{O}. The size and shape of the observed particles are compatible with tetramers. **C:** 2D class averages of 1380 particles of negative stain data representing smaller particles, likely dimers and monomers.

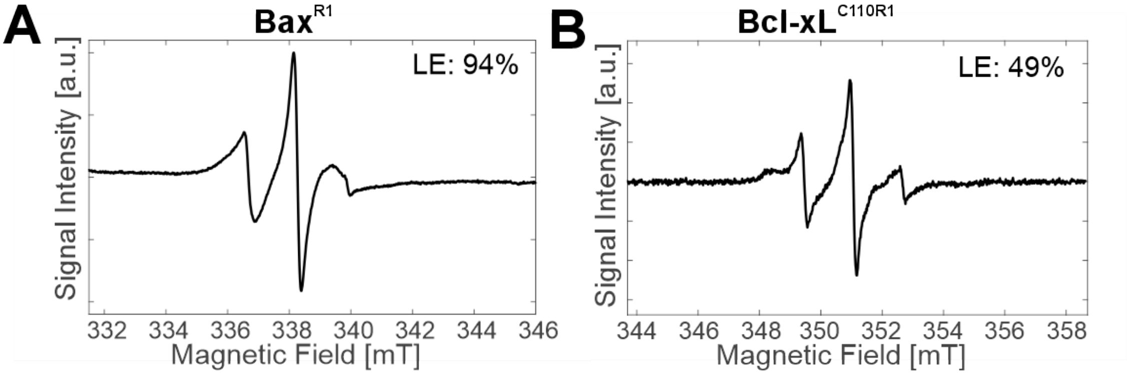

Figure S5: X-band cw EPR spectra. Measurements were performed at room temperature, labeling efficiencies (LE, calculated as spin per cysteine) of Bax^{R1} (**A**) and Bcl-xL^{C110R1}(**B**) were calculated using the double integral of the spectra and the protein concentration obtained by UV-vis.

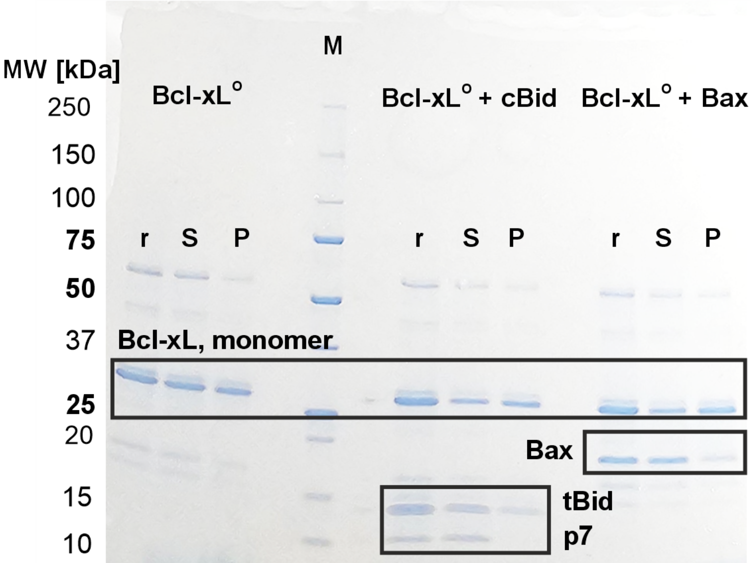

Figure S6: Analysis of water-membrane partitioning of oligomeric Bcl-xL. SDS PAGE analysis of Bcl-xL^{O} alone or in presence of cBid or Bax and LUVs. *P*: pellet obtained by the mixture with LUVs; *S*: supernatant obtained by the mixture with LUVs; *r*: reference protein mixture in solution without LUVs. Boxes highlight bands corresponding to monomeric Bcl-xL (26 kDa, upper panels), Bax (21 kDa, middle panel), cBid (tBid and p7 bands at 15 and 7 kDa, respectively, bottom panels). SDS PAGE samples were mixed with Laemmli buffer and DTT and heated to 95°C for 5 minutes.
